# Impact of technical aspects of vein of Marshall ethanol infusion on mitral isthmus block for persistent atrial fibrillation ablation

**DOI:** 10.3389/fcvm.2022.1031673

**Published:** 2022-10-04

**Authors:** Lingcong Kong, Tian Shuang, Zheng Li, Zhiguo Zou, Jun Pu, Xin-Hua Wang

**Affiliations:** Department of Cardiology, Renji Hospital, Shanghai Jiao Tong University School of Medicine, Shanghai, China

**Keywords:** ethanol infusion, vein of Marshall, persistent atrial fibrillation, radiofrequency catheter ablation, technical aspects

## Abstract

**Aims:**

Ethanol infusion into the VOM (EIVOM) adjunctive to radiofrequency catheter ablation (RFCA) was a novel approach facilitating mitral isthmus (MIth) block for persistent atrial fibrillation (PeAF); However, there were remarkable disparities in its technical aspects. This study aimed to evaluate the impact of EIVOM technical aspects on acute MIth block.

**Methods:**

Eighty consecutive patients (63 males, average age 66.4 ± 8.6 years) undergoing *de novo* PeAF ablation were assigned to different groups. The procedural parameters in “EIVOM first” (*n* = 13) or “RFCA first” (*n* = 13) as well as small dose ([SD], ≤4 ml, *n* = 26) or big dose ([BD], >4 ml, *n* = 54) approaches were analyzed to identify the predictors for acute MIth block.

**Results:**

Compared with the “EIVOM first” approach, the “RFCA first” approach was associated with longer procedural and MIth ablation time (134 ± 27 min vs. 112 ± 17 min; 14.9 ± 5.5 min vs. 9.3 ± 5.1 min, both *P* < 0.05, respectively), but with comparable success of MIth block. The ethanol dose was 6.3 ± 1.5 ml in BD group vs. 3.1 ± 1.0 ml in SD group (*P* < 0.001) and was correlated significantly with the size of Δlow voltage area (*r* = 0.66, *P* < 0.001). The success of MIth block was 92.6% in BD group vs. 73.1% in SD group, *P* = 0.03. The ethanol dose >5.75 ml independently predicted successful MIth block (OR: 0.428, 95% CI: 0.219–0.839, *P* = 0.01).

**Conclusions:**

Despite the comparable effectiveness on MIth block, the “EIVOM first” approach was associated with shorter procedural and MIth ablation time than the “RFCA first” approach. The ethanol dose in EIVOM was an independent predictor for MIth block.

## Introduction

Linear ablation is a commonly used approach of substrate modification for persistent atrial fibrillation (PeAF) (1). Mitral isthmus (MIth) ablation is technically challenging due to the complex anatomic structure, the “heat sink” effect of the blood flow of the circumflex and the coronary sinus (CS) (2, 3), and the epicardial connections of the CS and the vein of Marshall (VOM) bypassing the endocardial lesions (4). The surrounding myocardial bundles of VOM are frequently insulated by interposed fibrofatty tissue which is resistant to heat penetration, forming the epicardial connections in MIth ablation (5). The VOM is also an important non-PV AF source due to the myocardial bundles harboring AF triggers (6), and the surrounding autonomic innervation which might contribute to AF maintenance (7).

Dehydrated ethanol infusion into the VOM (EIVOM) is a novel approach initially proposed to facilitate left PV isolation (8). Subsequently it was found that additional EIVOM was associated with markedly shorter MIth ablation time, higher rate of MIth block (9, 10) and lower rate of recurrent atrial tachyarrhythmias (ATa) (11–13), compared with radiofrequency catheter ablation (RFCA) alone. Despite its consistently positive role in PeAF ablation in numerous studies, EIVOM could only be achieved in about 80.3–92% (11–13) of patients due to anatomical limitations; Furthermore, there were remarkable disparities with respect to ethanol dose, balloon position and timing of EIVOM relative to RFCA. In the earliest studies, a total of 2–4 ml ethanol was injected, with 1–2 ml over 2 min and 2 min apart (6, 9). Later on, a total of 9–12 ml ethanol was injected with 3 ml over 1 min for each infusion (11–13). The over-the-wire (OTW) balloon was either positioned distally for the first infusion and gradually retracted to the proximal VOM for sequential infusions (9, 13), or at the fixed site of the proximal VOM during all infusions in different studies (10, 14). EIVOM was the first step of ablation in most studies (9–14), while it was performed following RFCA in a few studies (6). It is not known whether or not the difference in technical aspects has impact on the procedural results.

This present study aimed to evaluate the impact of EIVOM technical aspects on acute MIth block in a cohort of patients undergoing *de novo* PeAF ablation.

## Materials and methods

### Study population

Eighty patients undergoing *de novo* PeAF ablation in Renji Hospital, Shanghai Jiao Tong University School of Medicine were consecutively enrolled from April 2020 to Jan 2022. PeAF was defined as continuous AF > 7 days without intermittent presence of sinus rhythm. Long-standing PeAF was defined as AF sustaining for more than one year (15). Patients were eligible for enrollment if the inclusion criteria were met: age range 18–82 years; symptomatic PeAF unresponsive to >1 antiarrhythmic drug (AAD) therapy; left atrial diameter (LAD) <58 mm measured by transthoracic echocardiography (TTE); successful EIVOM during *de novo* ablation. Exclusion criteria were: left atrial thrombus detected by transesophageal or intracardiac echocardiography; decompensated congestive heart failure; history of catheter or surgical AF ablation; history of cardiac surgery; unable to provide written informed consent.

### Study design

In this retrospective study a fixed anatomic approach combining circumferential pulmonary vein isolation (CPVI) with additional linear ablation at the left atrial roof and the MIth was applied for treating PeAF. Both RF energy and EIVOM were utilized. In the EIVOM procedure, the dose of dehydrated ethanol conformed to the methodological evolution at the time when the patient was enrolled.

According to the time of enrollment and the dose of ethanol all the patients were allocated into two groups: the Small Dose (SD) group (*n* = 26) and the Big Dose (BD) group (*n* = 54). In the SDG group, ≤4 ml ethanol was injected either before or after RFCA for early subjects, at the discretion of the operator. If EIVOM was performed after RFCA, the timing of EIVOM was after endocardial ablation but before CS ablation in order not to occlude the ostium of the VOM. In the BD group, the EIVOM was performed prior to RFCA and the target dose of ethanol infusion was >4 ml for subsequent subjects. All the RFCA procedural parameters, in conjunction with those of the EIVOM procedure, were collected to analyze any significant correlation with acute MIth block.

### Electrophysiological study

The procedure was performed under conscious sedation and analgesia by continuous transvenous infusion of fentanyl and midazolam. After written informed consent was obtained, a 6F decapolar mapping catheter (Abbott Medical, USA) was positioned in the CS *via* the left femoral vein. Two Swartz sheathes (SL1 Fast-Cath™, Abbott Medical, USA) were introduced into the RA. The transseptal procedure was performed under fluoroscopic or intracardiac echocardiography (CartoSound, Biosense Webster, USA) guidance. After the first transseptal puncture, unfractionated heparin 100 U/kg was injected and added 1,000 U every one hour to maintain an activated clotting time (ACT) range 300–350. A duo-decapolar mapping catheter (PentaRay, Biosense Webster, USA) was advanced in the LA through one Swartz sheath for PV potential recording and LA geometry reconstruction. The other Swartz sheath was used for guiding the EIVOM procedure and placing the ablation catheter in the LA for RF ablation.

### The ethanol infusion into the vein of Marshall procedure

A 6F coronary angiographic guiding catheter JR 4.0 (Judkins R4.0, Medtronic Inc, USA) was positioned into the distal CS through the Swartz sheath. Retrograde unselective venography was performed by manual contrast infusion to identify the VOM ostium. Care was taken to avoid CS dissection when manipulating the catheter. Subsequently, the guiding catheter was engaged to the VOM ostium, through which a 0.014 inch × 180 cm guidewire (Runthrough NS, Terumo, Japan) was gently advanced to the distal VOM as far as possible. Under the support of the guidewire an OTW balloon of proper size (1.5–2.5 mm × 8–12 mm, Emerge ™, Boston Scientific, USA) was advanced into the VOM.

**FIGURE 1:**
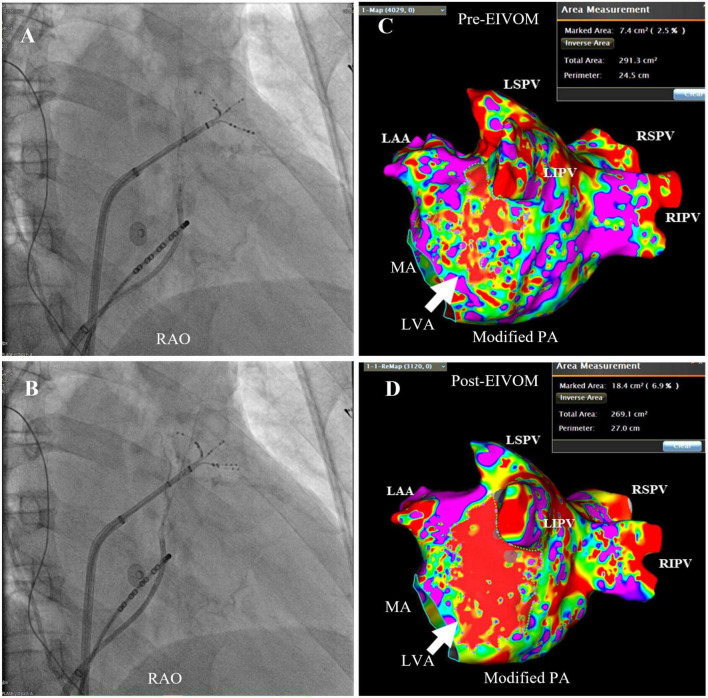
Selective venography of the EIVOM and LVA measurement. **(A)** The balloon at the proximal VOM was inflated at 7 atm, and selective venography displayed one small tortuous VOM with two distal branches, coursing upward behind the ostium of the LAA (the PentaRay catheter at the LAA). While in panel **(B)**, after balloon inflation at 8 atm, repeated venography showed another long and straight proximal branch of VOM [invisible in panel **(A)**], indicating the balloon was completely occluded in panels **(B)** and not in panel **(A)**. This example highlighted the importance total occlusion of the OTW before ethanol infusion. **(C,D)** LA substrate mapping pre- and post- EIVOM. Note the size of LVA (white arrow) markedly increased from 7.4 cm^2^ pre-EIVOM to 18.4 cm^2^ post-EIVOM. RAO, right anterior oblique; PA, postero-anterior; LSPV, left superior pulmonary vein; RSPV, right superior pulmonary vein; LIPV, left inferior pulmonary vein; RIPV, right inferior pulmonary vein; LAA, left atrial appendage; MA, mitral annulus; LVA, low voltage area.

In this study, the OTW balloon was fixedly placed at the proximal VOM. After inflation of the balloon at 6–8 atm, selective venography was performed to confirm total VOM occlusion (Figures 1A,B), display the arborization of the VOM and identify collateral branches communicating with other structures. The morphology of VOM was classified to three types referred to previous study (14): Type 1 as a slim trunk with multiple distal branches, type 2 as a large trunk with branches and type 3 as straight VOM without visible branches (Supplementary Figure 1). In early cases, successive 2 times of ethanol was injected with 1–2 ml for each infusion and 2 min apart. In subsequent cases, successive 2–3 times of ethanol was injected with 2–3 ml for each and 2 min apart. The infusion rate was less than 3 ml ethanol per minute. At the end of EIVOM, repeated venography was applied through the lumen of the balloon to observe tissue staining and exclude contrast extravasation into the free pericardium.

### Evaluation of left atrium substrate before and after the ethanol infusion into the vein of Marshall procedure

To evaluate the effect of ethanol infusion on LA scar formation, high density mapping was performed before and after the EIVOM procedure. The PentaRay catheter roved slowly in the LA to reconstruct the LA geometry as well as acquire 200–3,000 points evenly distributed at every aspect of the LA. Low voltage area (LVA) was defined as local bipolar peak-to-peak potential ≤0.3 mV during sustaining AF or ≤0.5 mV in NSR (Figures 1C,D) (10, 16), and was displayed as a red zone on LA geometry. The LVA contour was drawn manually and the LVA size was calculated by the built-in software tool of CARTO 3 system (Biosense Webster, USA). ΔLVA was defined as the difference between the size of LVA after and before EIVOM.

### Persistent atrial fibrillation ablation and the procedural endpoint

**FIGURE 2:**
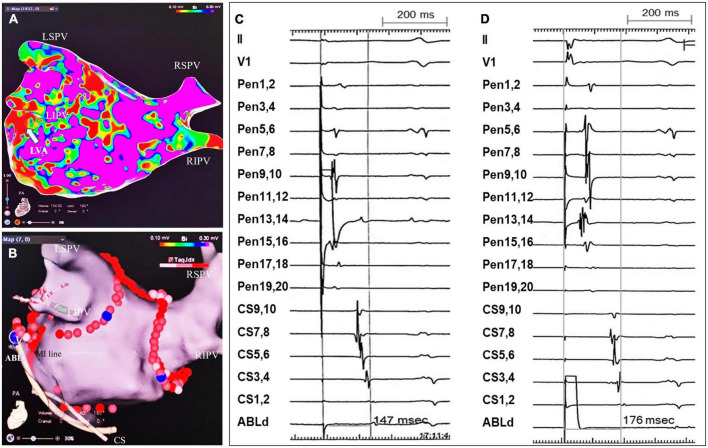
An example of MIth linear ablation and validation of MIth block. **(A)** LA substrate mapping post-EIVOM displayed a patchy LVA (white arrow) in the MIth region. **(B)** An entire MIth lesion line was created connecting the MA and the inferior aspect of LIPV’s ostium, across the EIVOM-related LVA. Note epicardial CS ablation was applied after failure of MIth block by endocardial MIth ablation. **(C,D)** Tracings were surface ECG lead II, V_1_, PentaRay_1_,_2_ -PenraRay_19_,_20_, CS_9_,_10_ - CS_1,2_ and ABL_1_,_2_. **(C)** Pacing from PentaRay_13_,_14_ (positioned within LAA) displayed the proximal to distal CS activation sequence. The interval from LAA pacing to CS1,2 WAS 146 milliseconds (ms). **(D)** The ablation catheter tip (ABL) was positioned just lateral to the MIth line [shown in panel **(B)**]. The interval from pacing from ABL_1_,_2_ to CS_1_,_2_ was 176 ms, which was longer than that from pacing from LAA to CS1,2 (146 ms), indicating the MIth line was blocked. LSPV, left superior pulmonary vein; RSPV, right superior pulmonary vein; LIPV, left inferior pulmonary vein; RIPV, right inferior pulmonary vein; LVA, low voltage area; MIth, mitral isthmus; CS, coronary sinus.

A 3.5 mm saline-irrigated contact-force sensing catheter (Thermocool SmartTouch^®^ SF, Biosense Webster, USA) was applied for creating RF lesions. For CPVI, RF energy was delivered at 40–45 W, saline irrigation speed 15 ml/min to achieve an ablation index (AI) 450–500 for anterior and roof lesions and 380–400 for posterior lesions. The endpoint of CPVI was disappearance of PV potentials (PVP) or dissociation of PVP with atrial electrograms in all PVs. For linear ablation, RF energy was delivered at 35–40 W, saline irrigation speed 15 ml/min and target AI 450–550 for endocardial ablation, and at 25 W, 30 s and saline irrigation speed 30 ml/min for epicardial ablation in the CS. An entire MIth lesion line was created connecting the lateral mitral annulus (MA) to the ostium of left inferior PV (LIPV) in all patients (Figures 2A,B). However, if RF lesions were created at the EIVOM-related LVAs (typically located at the anteroinferior antrum of the LIPV and the MIth), a lower target AI at 400–450 was set to reduce the risk of tissue overheating.

The endpoint of linear ablation was acute bidirectional block across the lines after 20 min observation, which was validated by differential pacing maneuvers.

Roof line block was deemed if: (1) Caudal to cranial conduction at LA posterior wall in NSR (17); (2) The interval from left atrial appendage (LAA) pacing to high posterior LA was longer than that to medium and low posterior LA; (3) the interval from high posterior LA pacing to LAA was longer than that from medium and low posterior LA pacing to LAA.

**FIGURE 3:**
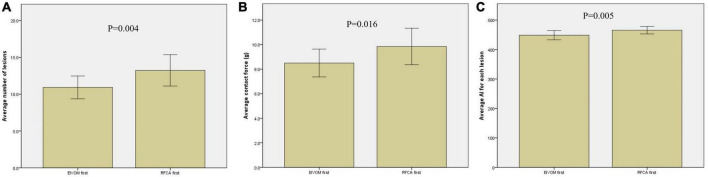
Comparison of RF ablation parameters between the “EIVOM first” and “RFCA first” group. The average ablation index (AI) **(A)**, contact force **(B)**, and number of lesions **(C)** was significantly greater in “RFCA first” group than those in “EIVOM first” group.

MIth block was considered if: (1) The proximal to distal CS activation by pacing from the LAA and the site just lateral to the lesion line (the distal electrode pair of CS catheter positioned just septal the lesion line); (2) The interval from pacing at the site just lateral the lesion line to CS_1,2_ was longer than that from LAA pacing to CS_1,2_ (Figures 2C,D); (3) The interval from CS_1,2_ pacing to the LAA was longer than that from CS_3,4_ and CS_5,6_ pacing to the LAA (18).

### Follow-up

TTE was performed immediately after the procedure and repeated, if necessary, after ablation to rule out pericardial effusion. Acute pericarditis was considered if chest pain aggravated by deep inspiration within one-week post-ablation. All the patients were kept on oral anticoagulation with novel oral anticoagulants (NOACs) for at least 3 months. Amiodarone or other Class IC AADs were administered for 3 months. They were followed up regularly at the outpatient clinic 1-, 3-, 6-, 12 months post-ablation or whenever experienced symptoms indicating a recurrence for the recording of ECGs and 24-h Holter monitoring. After the blanking period of 3 months, any documented episode of ATa lasting more than 30 s was considered as a recurrence. If no ATa recurrence was detected, AADs could be discontinued. Persistence of oral anticoagulation depended on the evaluation of thromboembolic risks in each patient.

### Statistical analysis

Continuous data of normal distribution were described as mean ± SD or as median (1st quartile, 3rd quartile) otherwise. Student’s *t*-test was applied to compare continuous data if the variance was equal, otherwise Mann–Whitney *U* test as appropriate. Category data were given as counts or proportions, and compared by Chi-square test or Fisher’s exact test. The association of ethanol dose with the size of ΔLVA was tested by Spearman’s correlation test. Variables with *P* value <0.10 in univariate analysis were included in multivariate analysis. The predictors for MIth block were evaluated by multivariate binary logistic regression analysis [odds ratio (OR) and 95% confidence interval (CI)]. A receiver operating characteristic (ROC) curve was calculated to determine the area under the curve (AUC), sensitivity and specificity of the predictors, and assess the cut-off value that predict MIth block. The cumulative ATa-free survival probability was assessed by Kaplan-Meier analysis and compared by Log-rank test. A two-tailed *P* value <0.05 was considered statistically significant. Statistical analysis was performed by SPSS 26.0 software (IBM Corporation, Somers, NY, USA).

## Results

### Patients’ demographic data

Eighty patients (63 males, average age 66.4 ± 8.6 years) with PeAF successfully underwent RF ablation and adjunctive EIVOM procedure. The median AF duration was 11.5 months. The average LAD was 48.0 ± 4.3 mm, and LVEF <50% was present in 11 patients. There were 14 patients with history of stroke, 16 with history of LAA device closure. The demographic data were shown in Table 1.

**TABLE 1:** Demographic data of the patients enrolled in this study.

Parameters	Value
Cases (*N*)	80
Age (years)	66.4 ± 8.6
Male, *n* (%)	63 (78.8)
BMI (m/kg^2^)	25.2 ± 3.5
Duration of AF (months)	11.5 (2.0, 36.0)
Number of AADs used	1.5 ± 0.8
CHA_2_DS_2_-VASc score	2.6 ± 1.4
**Comorbidities**	
Hypertension, *n* (%)	59 (73.7)
Diabetes mellitus, *n* (%)	10 (12.5)
Coronary artery disease, *n* (%)	7 (8.8)
Dilated cardiomyopathy, *n* (%)	7 (8.8)
Patients with LVEF < 50%, *n* (%)	11 (13.8)
History of stroke, *n* (%)	14 (17.5)
History of LAAC, *n* (%)	16 (20)
**TTE parameters**	
LAD (mm)	48.0 ± 4.3
LVEDD (mm)	49.9 ± 5.7
LVESD (mm)	34.2 ± 6.1
LVEF (%)	57.8 ± 9.7

BMI, body mass index; AAD, antiarrhythmic drug; LAD, left atrial diameter; LVEDD, left ventricular end diastolic diameter; LVESD, left ventricular end systolic diameter; LVEF, left ventricular ejection fraction; LAAC, left atrial appendage closure.

### Comparison between “radiofrequency catheter ablation first” and “ethanol infusion into the vein of Marshall first” in the small dose group

In the SD group, there were 13 patients undergoing “RFCA first” ablation and 13 patients undergoing “EIVOM first” ablation. There was significantly longer procedural time and RF ablation time at the MIth, as well as smaller ethanol dose and the size of ΔLVA in “RFCA first” sub-group than those in “EIVOM first” sub-group. Right and left PV isolation time, proportion of roof line block, proportion of CS ablation, VOM morphological features, ethanol infusion times/duration and the success rate of MIth block were comparable in both sub-groups (Table 2).

**TABLE 2:** Comparison of the procedural parameters between different groups.

Procedural parameters	Total (*n* = 80)	EIVOM first (*n* = 13)	RFCA firs (*n* = 13)	SD group (*n* = 26)	BD group (*n* = 54)
Procedural time (min)	128 ± 24	112 ± 17*	134 ± 27*	124 ± 25	130 ± 23
RPV ablation time (min)	20.2 ± 5.6	21.5 ± 4.5	23.9 ± 6.0	22.5 ± 5.4	20.2 ± 5.5
LPV ablation time (min)	17.3 ± 7.2	19.0 ± 10.4	21.9 ± 9.7	20.5 ± 10.0^#^	15.8 ± 4.9^#^
Proportion of PVI, *n* (%)	80 (100)	13 (100)	13 (100)	26 (100)	54 (100)
RF ablation time at the MIth (min)	12.7 ± 5.7	9.3 ± 5.1*	14.9 ± 5.5*	12.1 ± 5.9	13.0 ± 5.6
Ablation in the CS, *n* (%)	52 (65)	9 (69.2)	7 (53.8)	16 (61.5)	36 (66.7)
Proportion of roof line block, *n* (%)	59 (73.8)	11 (84.6)	9 (69.2)	20 (76.9)	39 (72.2)
Number of lesions of MIth	13 ± 3	11 ± 2*	13 ± 2*	12 ± 2	13 ± 4
AI for each lesion of MIth	446 ± 17	449 ± 16*	466 ± 13*	457 ± 16^$^	440 ± 15^$^
Contact force of MIth (g)	8.7 ± 1.5	8.5 ± 1.1*	9.8 ± 1.5*	9.2 ± 1.5	8.5 ± 1.5
Total amount of energy of MIth (kJ)	27.5 ± 12.7	19.6 ± 10.7*	35.9 ± 13.5*	27.8 ± 14.6	27.4 ± 11.8
Type 1 VOM, *n* (%)	66 (82.5)	10 (76.9)	11 (84.6)	21 (80.8)	45 (83.3)
Type 2 VOM, *n* (%)	1 (1.2)	0 (0)	0 (0)	0 (0)	1 (1.9)
Type 3 VOM, *n* (%)	13 (16.3)	3 (23.1)	2 (16.3)	5 (19.2)	8 (14.8)
Diameter of VOM trunk (mm)	2.0 ± 0.5	2.0 ± 0.4	2.0 ± 0.3	2.0 ± 0.4	2.0 ± 0.5
Number of VOM branches	2.1 ± 1.2	1.8 ± 1.2	2.1 ± 1.2	2.0 ± 1.2	2.1 ± 1.2
Total ethanol dose (ml)	5.3 ± 2.0	3.5 ± 0.8*	2.7 ± 1.0*	3.1 ± 1.0^$^	6.3 ± 1.5^$^
Injection times	2.4 ± 1.0	1.6 ± 0.5	1.4 ± 0.5	1.5 ± 0.5^$^	2.9 ± 0.8^$^
EIVOM duration (s)	480 (300, 660)	240 (60, 300)	240 (150, 300)	240 (60, 300)^#^	240 (150, 300)^#^
ΔLVA (cm^2^)	5.0 (3.3, 7.1)	4.0 (2.2, 7.3)*	1.2 (0.0, 4.8)*	3.4 (0.4, 5.4)^$^	5.6 (4.3, 7.8)^$^
Proportion of MIth block, *n* (%)	69 (86.3)	11 (84.2)	8 (61.5)	19 (73.1)^‡^	50 (92.6)^‡^

*Indicated *P* < 0.05 for comparison between “EIVOM first” and “RFCA first” in SD Group. ^#^Indicated *P* < 0.01 for comparison between SD group and BD group. ^$^Indicated *P* < 0.001 for comparison between SD group and BD group. ^‡^Indicated *P* < 0.05 for comparison between SD group and BD group. RPV, right pulmonary vein; LPV, left pulmonary vein; CS, coronary sinus; MIth, mitral isthmus; AI, the ablation index; LVA, low voltage area; RF, radiofrequency.

Comparison of RF ablation at the MIth in sub-groups: Besides longer RF ablation time at the MIth and similar proportion of CS ablation (Table 2), there was significantly greater number of lesions, higher average ablation index (AI), higher average contact force and larger total amount of energy in “RFCA first” than those in “EIVOM first” sub-group (Table 2 and Figure 3).

### Comparison between the procedural parameters in the small dose group and big dose group

There were 26 patients in the SD group and 54 patients in the BD group. There was significant difference in left PV isolation time, total ethanol dose, EIVOM infusion times/duration and size of ΔLVA between the SD and BD group. The success rate of MIth block was significantly higher in the BD group than that in the SD group (92.6 vs. 73.1%, *P* = 0.03). Total procedural time, right PV isolation time, RF ablation time at the MIth, proportion of CS ablation, proportion of roof line block and VOM morphological parameters were comparable in two groups (Table 2).

**FIGURE 4:**
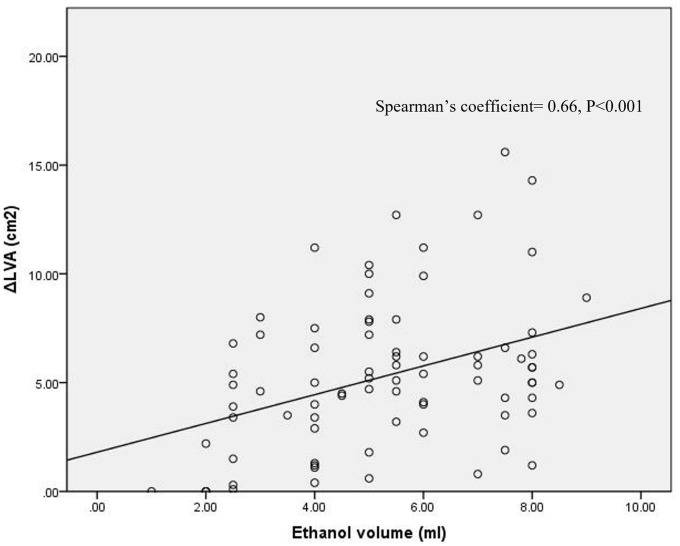
Correlation of ethanol volume with the size of ΔLVA at the MIth region. Spearman’s coefficient = 0.66, *P* < 0.001.

In all 80 patients from SD and BD group, the ethanol dose correlated significantly with the size of ΔLVA (ΔLVA = 0.66 × ethanol dose + 1.806, *P* < 0.001), shown in Figure 4.

### Comparison between successful and failed mitral isthmus block in the patients undergoing “ethanol infusion into the vein of Marshall first” ablation

Acute MIth block was achieved in 61 patients and not in the remaining 6 in total 67 patients undergoing “EIVOM first” ablation. The demographic characteristics (including age, gender, CHA_2_DS_2_-VASc score, AF duration and TTE measurement) and proportion of roof line block were comparable in two groups, while the total procedural time, left PV ablation time and RF ablation time at the MIth was significantly shorter, and the ethanol dose/the size of ΔLVA was greater in successful MIth block group than those in failed MIth block group. The mean interval from LAA pacing to distal CS was 162.8 ± 33.7 ms after successful MIth block, as compared with 97.2 ± 32.9 ms for failed MIth block (Table 3).

**TABLE 3:** Comparison between successful and unsuccessful MIth block in the EIVOM first approach.

Factors	Successful MI block (*n* = 61)	Unsuccessful MI block (*n* = 6)	*P*-value
Age (years)	67.1 ± 8.0	69.0 ± 6.6	0.58
Male, *n* (%)	47 (77.0)	6 (100)	0.33
BMI (m/kg^2^)	24.9 ± 3.4	24.8 ± 3.2	0.92
LAD (mm)	48.0 ± 4.6	48.8 ± 3.4	0.65
CHA_2_DS_2_-VASc score	2.5 ± 1.4	2.8 ± 1.3	0.63
Duration of AF (months)	12 (2, 36)	32 (1, 141)	0.72
Procedural time (min)	124.8 ± 22.5	147.5 ± 20.7	0.02
RPV ablation time (min)	19.5 ± 5.4	20.0 ± 5.1	0.84
LPV ablation time (min)	15.7 ± 4.7	24.0 ± 14.4	0.001
RF ablation time at the MIth (min)	11.7 ± 5.1	19.0 ± 7.7	0.002
Ablation in the CS, *n* (%)	37 (60.7)	6 (100)	0.08
Proportion of roof line block, *n* (%)	45 (73.8)	5 (83.3)	0.61
Type 1 VOM	50 (82.0)	5 (83.3)	0.95
Type 2 VOM	1 (1.6)	0 (0)	
Type 3 VOM	10 (16.4)	1 (16.7)	
Diameter of VOM trunk (mm)	2.0 ± 0.5	2.0 ± 0.6	0.96
Number of VOM branches	2.0 ± 1.2	2.7 ± 1.8	0.24
Total ethanol dose (ml)	6.0 ± 1.7	3.8 ± 1.3	0.004
Injection times	2.7 ± 0.9	2.2 ± 1.0	0.20
EIVOM duration (s)	525 (300, 660)	300 (250, 660)	0.23
ΔLVA (cm^2^)	5.4 (3.8, 7.7)	4.9 (3.0, 6.8)	0.01
LAApacing-CSd interval (ms)	162.8 ± 33.7	97.2 ± 32.9	<0.001

BMI, body mass index; LAD, left atrial diameter; RPV, right pulmonary vein; LPV, left pulmonary vein; CS, coronary sinus; LVA, low voltage area; RF, radiofrequency; MIth, mitral isthmus; LAA, left atrial appendage.

### Predictors for acute mitral isthmus block in the “ethanol infusion into the vein of Marshall first” approach

**FIGURE 5:**
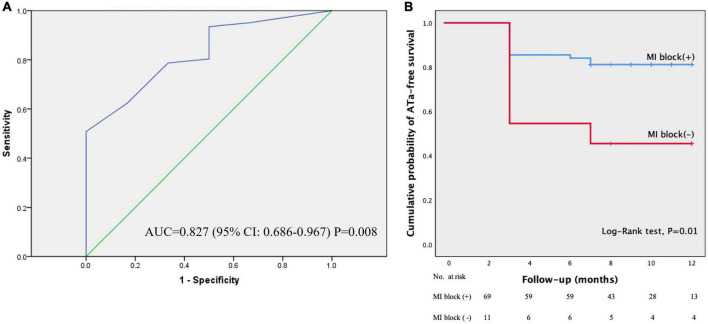
The ROC curve for the ethanol volume and comparison of ATa-free survival. Panel **(A)** displayed the ROC curve for the ethanol volume. Area under the curve (AUC) was 0.827, 95% CI: 0.686–0.967, *P* = 0.008. Panel **(B)** showed the Kaplan–Meier plots of ATa-free survival in patients with MIth block and those without during one year’s follow-up. The ATa-free survival was significantly higher in patients with successful MIth block than that in those without (Log-rank test, *P* = 0.01). ATa, atrial tachyarrhythmias.

Of all the baseline and procedural parameters, binary Logistic regression analysis showed that total ethanol dose was the only independent predictor for acute MIth block in a “EIVOM first” approach (OR: 0.428, 95% CI: 0.219–0.839, *P* = 0.01). ROC analysis showed the cut-off ethanol dose for predicting MIth block was 5.75 ml (sensitivity 50.8% and specificity 100%; AUC = 0.827, 95% CI: 0.686–0.967, *P* = 0.008) (Figure 5A).

### Complications

Transient atrioventricular block occurred in 1 case when inadvertently manipulating the guiding catheter close to the atrioventricular node and recovered several hours post-ablation. Despite CS dissection occurring in 1 case when introducing the guiding catheter into the CS, no evidence of contrast exosmosis was observed and the EIVOM was completed without any sequelae. Trivial pericardial effusion was noted in 2 cases at 48 h post-ablation but need not further management. Pericarditis in 3 cases 1–2 days post-ablation subsided in one week without any drug treatment. No cardiac tamponade was detected at the peri-procedural period of 30 days post-ablation.

### Follow-up results

After a mean follow-up of 9.7 ± 1.9 (range 7–12) months, 61 patients were free of ATa recurrence off AADs. In the remaining 19 patients, ATa recurred at a mean of 3.8 ± 1.6 months post-ablation, including AF in 12 patients and atrial flutter in 7 patients. Compared with the patients without MIth block (*n* = 11), those with successful MIth block (*n* = 69) had significantly higher ATa-free survival probability (Log-rank test, *P* = 0.01) (Figure 5B).

## Discussion

This retrospective study was of among the earliest studies focusing on the impact of the detailed technical aspects of EIVOM on acute MIth block, one of the most important procedural endpoints in an anatomic approach for PeAF ablation.

**FIGURE 6:**
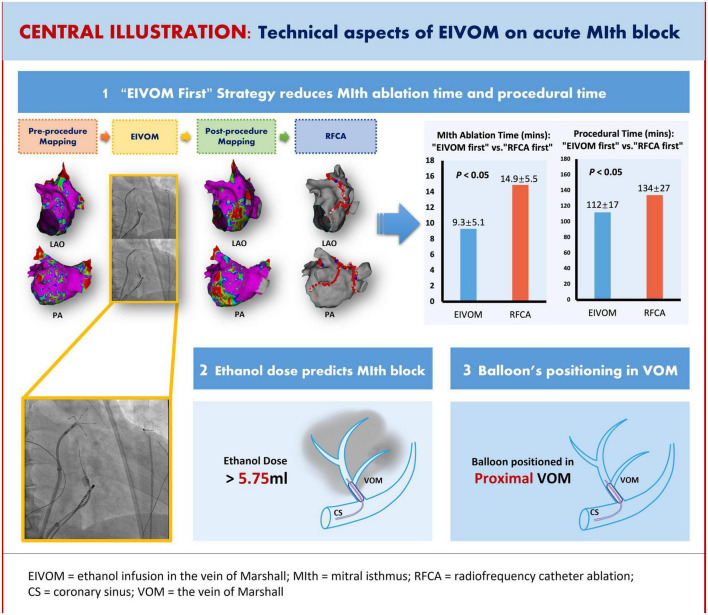
Central illustration: Technical aspects of EIVOM on acute MIth block.

The “EIVOM first” and the “RFCA first” approach were equally effective for achieving MIth block, although the latter needed longer procedural time and RF ablation time at the MIth. In contrast to small dose EIVOM (either before or after FRCA) approach, big dose EIVOM (before RFCA) approach was associated with higher success rate of MIth block and shorter LIPV isolation time. Of all the baseline and procedural parameters, the ethanol dose used in EIVOM (cut-off value 5.75 ml) was the only independent predictor for MIth block (Figure 6).

### The timing of ethanol infusion into the vein of Marshall and the superiority of the “ethanol infusion into the vein of Marshall first” approach

The “EIVOM first” approach was associated with higher first-pass success of MIth block compared with the “RFCA first” approach; However, the final success of MIth block was comparable after touch-up RF ablation (19). The results of our study were partially in line with these findings. However, we created an entire MIth line rather than touch-up ablation at the residual channel. The rationale of this approach was based on the fact that the transmural property of epicardial ethanol infusion was not supported by sufficient data. The lesion quality might be compromised if the ethanol dose was insufficient. Another issue was the timing of EIVOM relative to epicardial RF ablation. The rate of failed VOM visualization was significantly higher in “RFCA first” than in “EIVOM first” (30 vs. 9%) (19) due to inadvertent occlusion the ostium of the VOM. For this reason, we suggest EIVOM be performed before CS ablation in the “RFCA first” subgroup to avoid this inconvenience.

Compared with the “RFCA first” approach, the “EIVOM first” approach was associated with shorter procedural and MIth ablation time without compromised safety, which contributed to establish an efficient and effective approach combining RFCA and EIVOM for PeAF ablation.

### Impact of ethanol dose on acute mitral isthmus block

It was well-known that the ethanol dose was found to correlated with LVA size (10), which was consistent with the finding in our study. However, the correlation of ethanol dose with the success of MIth block had not been reported to the best of our knowledge. In our study, we found the success rate of MIth block was higher in the BD group than those in the SD group. Further multivariate analysis showed that the dose of ethanol was the only significant predictor for MIth block. A cut-off value of 5.75 ml could predict MIth block with a high specificity and sensitivity. This new finding provided valuable information for the electrophysiological (EP) physicians when adopting an approach combining RFCA with EIVOM for PeAF ablation.

### Evolution of balloon positioning in the vein of Marshall procedure

In contrast to variable balloon positioning, Fixed balloon positioning might be equally efficacious but more advantageous for safety concerns: on one hand, similar to the distal VOM infusion, the ethanol could also reach the distal VOM by continuous infusion through the balloon at the proximal VOM; on the other hand, fixed balloon positioning helped to avoid frequent balloon inflation and deflation at the distal VOM, which was vulnerable to perforate, and avoid frequent contrast infusions for confirmation of complete occlusion of the VOM, which was prone to causing dissection. We suggested that mobile balloon positioning might be unnecessary in most cases except in rare cases with extremely huge VOMs (type 2 VOM).

### Other technical aspects of the ethanol infusion into the vein of Marshall procedure

It was reasonable that the types of VOM might have significant impact on MIth block, since the diameter and branches of the VOM might influence the total dose of ethanol injected in the VOM and eventually affect the size of the LVA. However, the results of our study did not support the VOM types were associated with success rate of MIth block. This might be explained by the fact that the total ethanol dose was not only dependent on the morphology of VOM, but also on the experience of the EP doctors.

To be noted, the VOM types might affect the success rate of MIth block in rare cases with VOMs shunting to other structures, because the remaining dose of ethanol was insufficient to create enough tissue damage.

### Limitations

This study had several major limitations. Firstly, this was a retrospective study and the number of patients enrolled was limited to compare the difference of “EIVOM first” and “RFCA first” ablation and to analyze the predictors for MIth block. Secondly, the criterion for dividing the patients into small dose and big dose group was arbitrary. However, the most appropriate ethanol dose used in the EIVOM procedure was still under investigation. The ethanol dose evolved from 1–4 ml in early stage to 9–12 ml in recent studies, which served as basis for patient grouping in this study. Thirdly, the repeated procedures were not included in this study and the durability of MIth block after ethanol infusion could not be evaluated. Lastly, the follow-up duration might be too short to evaluate the long-term effectiveness of adjunctive EIVOM on PeAF ablation.

## Conclusions

Despite the comparable effectiveness on MIth block, the “EIVOM first” approach was associated with shorter procedural and RF ablation time at the MIth than the “RFCA first” approach. The dose of ethanol used in EIVOM was an independent predictor for MIth block.

### Clinical perspectives

• Adjunctive EIVOM to RFCA facilitated mitral isthmus block for patients with persistent atrial fibrillation.
• In contrast to the “RFCA first” approach, the “EIVOM first” approach had shorter procedural time, RF ablation time at the MIth, and comparable MIth block success.
• The ethanol dose significantly correlated to the size of ΔLVA, and was a potent predictor for successful acute MIth block.

## Data availability statement

The raw data supporting the conclusions of this article will be made available by the authors, without undue reservation.

## Ethics statement

The studies involving human participants were reviewed and approved by the Renji Hospital Human Research Ethics Committee. The patients/participants provided their written informed consent to participate in this study.

## Author contributions

XW conceptualized the framework. XW, LK, and TS drafted the manuscript. LK and ZL involved in clinical data collection. ZZ involved in data processing. JP revised the manuscript. All authors have read and approved the final manuscript.

## Supplementary material

Supplementary Figure 1 The morphological categorization of VOM. Panel **(A)** displayed Type 1 VOM as a slim trunk (diameter ≤ JR 4.0) with multiple distal branches; Panel **(B)** illustrated type 2 VOM as a large trunk (diameter >JR 4.0) with branches; Panel **(C)** represented type 3 as straight VOM without visible branches. JR 4.0, Judkins R4.0 coronary angiographic guiding catheter.

## Abbreviations

AAD: antiarrhythmic drug
ATa: atrial tachyarrhythmias
CS: coronary sinus
CPVI: circumferential pulmonary vein isolation
EIVOM: ethanol infusion in the vein of Marshall
LA: left atrium
MIth: mitral isthmus
PeAF: persistent atrial fibrillation
RFCA: radiofrequency catheter ablation
VOM: the vein of Marshall.
