# Supplementary figures and images for: Impact of technical aspects of vein of Marshall ethanol infusion on mitral isthmus block for persistent atrial fibrillation ablation
Source: Front Cardiovasc Med. 2022 Oct 4;9:1031673. doi: 10.3389/fcvm.2022.1031673 (PMC9576952; doi:10.3389/fcvm.2022.1031673)

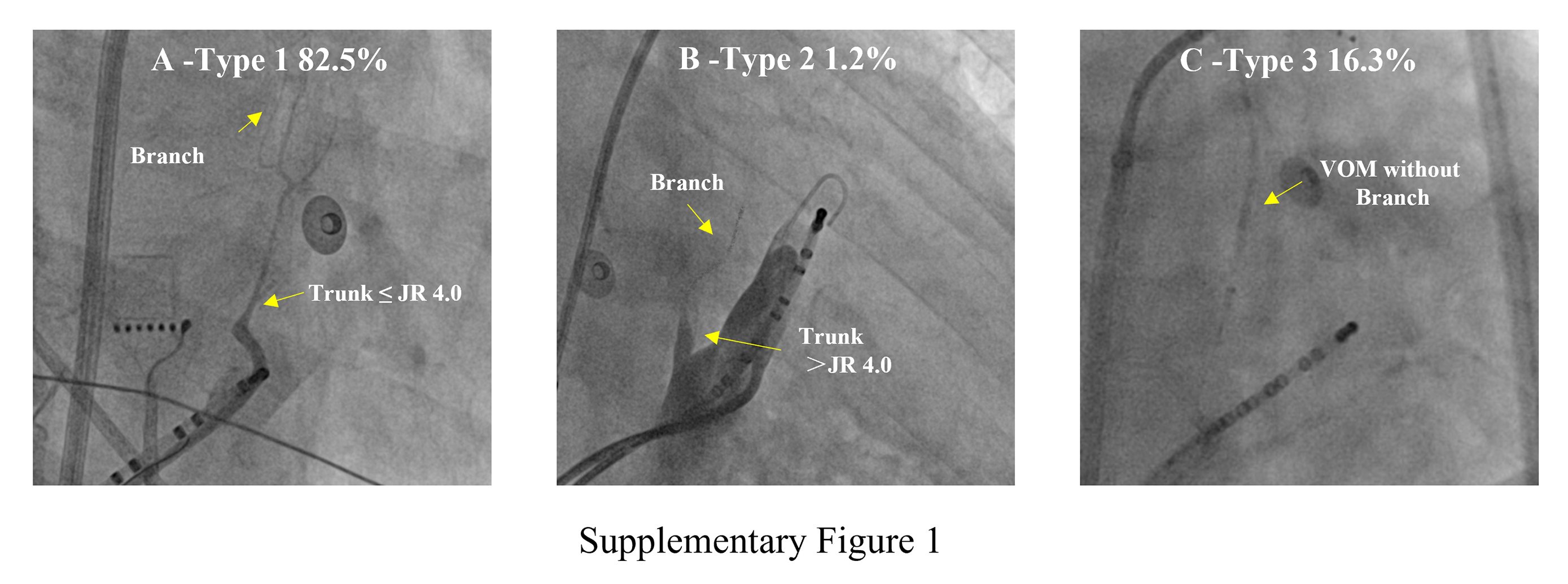

Supplement: Supplementary Figure 1 — The morphological categorization of VOM. Panel (A) displayed Type 1 VOM as a slim trunk (diameter ≤ JR 4.0) with multiple distal branches; Panel (B) illustrated type 2 VOM as a large trunk (diameter >JR 4.0) with branches; Panel (C) represented type 3 as straight VOM without visible branches. JR 4.0, Judkins R4.0 coronary angiographic guiding catheter. [file Image_1.tif]
